# *Hypericum Perforatum* L. Hypericaceae/Guttiferae Sunflower, Olive and Palm Oil Еxtracts Attenuate Cold Restraint Stress – Induced Gastric Lesions

**DOI:** 10.3390/molecules15106688

**Published:** 2010-09-28

**Authors:** Ivana Arsić, Ana Žugić, Dušanka Runjajić Antić, Gordana Zdunić, Dragana Dekanski, Goran Marković, Vanja Tadić

**Affiliations:** 1 Institute for Medicinal Plant Research, Dr Josif Pančić, Tadeuša Košćuška 1, 11 000 Belgrade, Serbia; E-Mails: azugic@mocbilja.rs (A.Ž.); drunjajic@mocbilja.rs (D.R.A.); gzdunic@mocbilja.rs (G.Z.); gmarkovic@mocbilja.rs (G.M.); vtadic@mocbilja.rs (V.T.); 2 Biomedical Research, R&D Institute, Galenika a.d., Pasterova 2, Belgrade, Serbia; E-Mail: ddekan@sezampro.rs (D.D.)

**Keywords:** *Hypericum perforatum*, sunflower, olive, palm oil, quercetin, gastroprotective activity

## Abstract

Three *Hypericum perforatum* L., Hypericaceae (St. John’s Wort) oil extracts (HPE) were prepared according to the prescriptions from traditional medicine – fresh flowering tops were macerated in three different vegetable oils: sunflower (E1), olive (E2) and palm oil (E3) for 40 days, exposed to the sunlight. The aim of the study was to investigate the gastroprotective activity of the obtained extracts in respect to their quercetin content. HPLC analysis confirmed the presence of quercetin in all of the investigated HPEs, but in different amounts: 15.1, 5.8 and 21.7 μg/mL in E1, E2, and E3, respectively. Gastroprotective activity was evaluated using cold-restraint stress (CRS) induced rat gastric mucosa lesions test. All of the HPEs showed gastroprotective activity, which was close to that achieved by the one of the most studied anti-ulcer flavonoids, quercetin [percentages of inhibition of ulcer index (UI) were 35, 62 and 40 % in E1, E2 and E3, respectively]. Contrary to the lowest quercetin content, HPE prepared with olive oil (E2) offered the highest protection against gastric damaging action of CRS. It may be assumed that this is due to other constituents of E2, which probably play an additional role in complex gastroprotective activity.

## 1. Introduction

*Oleum Hyperici* has been used as one of the oldest remedies in Serbian folk medicine in the treatment of cuts, burns, hemorrhoids and also as an antiseptic, for liver and stomach complaints, diarrhea, gastric ulcers, *etc*. It is obtained by maceration of the fresh flowering tops of *Hypericum perforatum* L. Hypericaceae (St. John’s Wort) in olive, sunflower or wheat-germ oil exposed to sunlight for 40 days [1,2]. According to literature data, utilization of *Oleum Hyperici* is known not only in Serbian traditional medicine, but also in other countries. As a matter of fact, similar methods for its preparation have been described in the supplement of German Pharmacopoeia [DAB 6] [3]. Moreover, German Commission E approved its usage for several indications, including treatment and post-therapy of acute and contused injuries, myalgia and first-degree burns as well as dyspeptic complaints [4].

Data regarding effects of St. John’s Wort on the gastric mucosa are controversial. Yesilada and Gürbüz reported a potent antiulcer activity of St. John’s Wort ethanol extract on the ethanol-induced gastric lesions in mice [5] while another study showed that St. John’s Wort inhibits gastric acid secretion in pyloric-ligated rats, but increases indomethacin-induced gastric mucosal lesions in a dose dependent manner [6]. Novel research has revealed that moderate doses of St. John’s Wort extracts heal hypothermic restraint stress-induced gastric ulcer in rats [7]. Data concerning *Oleum Hyperici* chemical composition and pharmacological activity are limited. Studies proved the presence of hyperforins and its analogues as well as of flavonoids, quercetin and I3,II8-biapigenin, but the absence of the naphthodianthrones (hypericin) [8,9], although Isacchi *et al* claimed only the presence of phloroglucinol derivates and I3,II8-biapigenin [10]. Investigation of St. John’s Wort oil preparations showed that a mixture of St. John’s Wort and *Calendula arvensis* oil extracts used externally had positive effects on epithelial reconstruction of surgical wounds [11]. In addition, traditionally claimed wound healing effect of St. John’s Wort olive oil extract was confirmed in the study of Süntar *et al* [12]. Zdunić *et al* investigated anti-inflammatory and gastroprotective activity of three St. John’s Wort extracts all prepared with sunflower oil but in three different ways. They provided evidence for the usage of these extracts as anti-inflammatory and gastroprotective agent and presumed that flavonoids identified in the extracts (quercetin and I3, II8-biapigenin) contribute to these activities [13].

For almost 30 years, our Institute has had its own trade marked product for the indications mentioned above. This product is sunflower oil extract of fresh St. John’s Wort flowering tops. The aim of the present study was to investigate quercetin content and gastroprotective activity of *Oleum Hyperici* prepared according to prescription from traditional medicine with three different types of extragenses: sunflower, olive and palm oil.

## 2. Results and Discussion

**Figure 1:**
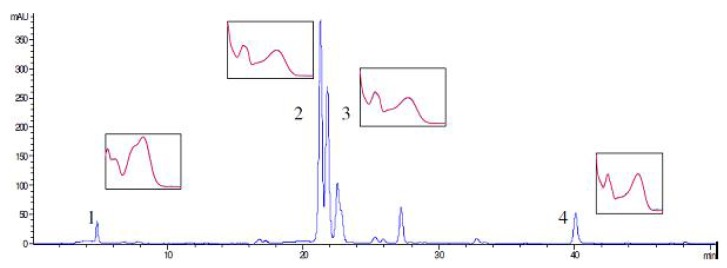
HPLC profile of St. John’s Wort methanolic extract: (1) chlorogenic acid, (2) rutin, (3) hyproside, (4) quercetin.

Chemical characterization of the plant material was carried out by HPLC method, for identification and quantification of flavonoids (rutin, hyperoside, quercetin) as well as chlorogenic acid, and spectrophotometric method for the total hypericins content determination. The results are given in **Table 1** and **Figure 1**. The presented results are in accordance with the chemical composition of St John’s Wort methanolic extract regarding the phenolic compounds content [14].

**Table 1:** Chemical analysis of St. John’s Wort methanolic extract.

*Compound*	*Method*	*Results*
Total hypericins	Spectrophotometry	0.47%
Rutin	HPLC	0.61%
Hyperoside	HPLC	0.78%
Quercetin	HPLC	0.02%
Chlorogenic acid	HPLC	0.03%

**Figure 2:**
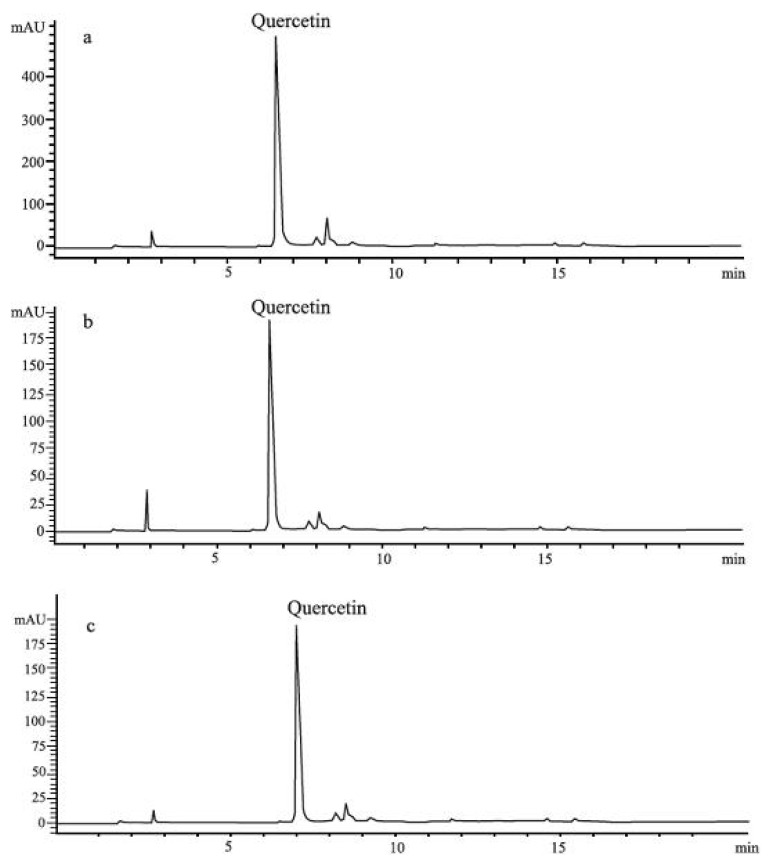
HPLC profiles of St. John’s Wort extracts: **(a)** E1, **(b)** E2, **(c)** E3.

In all investigated oil extracts HPLC analysis confirmed only the presence of quercetin, and we determined the quercetin content in *Oleum Hyperici* prepared according to the traditional medicine prescription with three different types of extragenses. However, its amount varied between samples (Figure 2). The lowest content of quercetin (5.8 μg/mL) was found in the sample prepared with olive oil (E2). In the sample E1, prepared with sunflower oil, the amount of quercetin was 15.05 μg/mL. The sample prepared with palm oil (E3) showed the highest level of quercetin (21.68 μg/mL).

Quantification of total hypericins in all investigated St. John’s Wort oil extracts (E1, E2 and E3) was also carried out. The results are given in Table 2. However, the relationship between naphtodianthrone content and the gastroprotective activity was not within the scope of the presented study.

**Table 2:** Total hypericins content in St. John’s Wort oil extracts.

*Sample*	*Hyp%*
E1	0.004%
E2	0.003%
E3	0.001%

**Figure 3:**
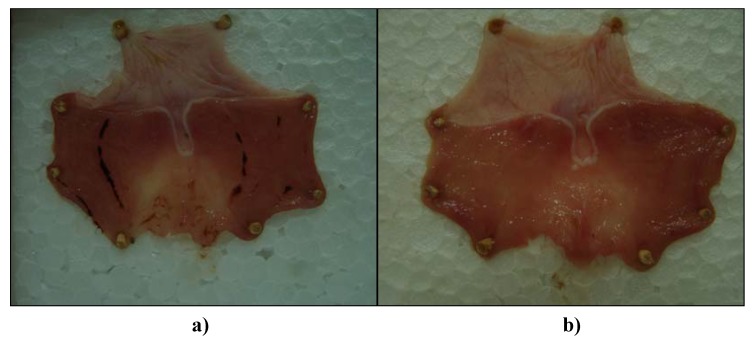
Representative rats' stomachs after cold restraint stress **(a)** control rat receiving sunflower oil (1.25 mL/kg) and **(b)** rat pretreated with St. John’s Wort olive oil extract (1.25 mL/kg).

**Figure 4:**
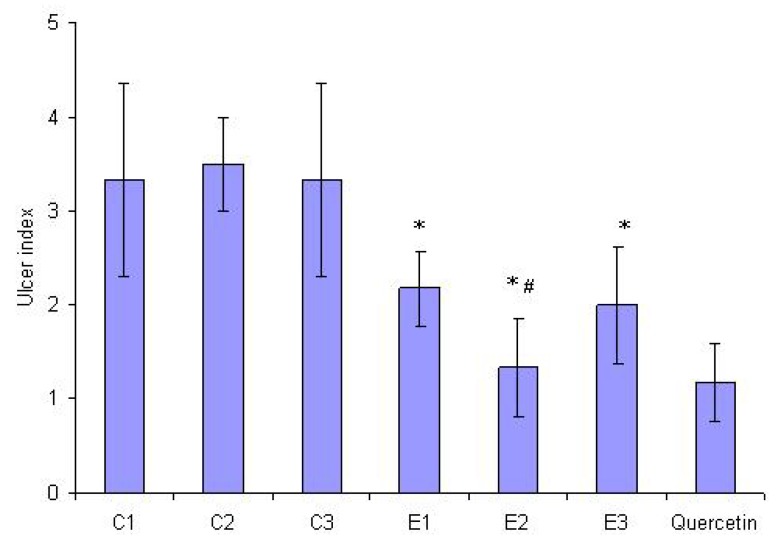
Effect on intragastric pretreatment with St. John’s Wort extracts E1, E2 and E3 (1.25 mL/kg) and quercetin (8 mg/kg) on the ulcer index induced by CRS. * indicates statistical significance in ulcer index, as compared to appropriate control value (C1, C2 and C3). # indicates statistical significance in ulcer index of E2, as compared to E1 and E3.

Cold-restraint stress (CRS)-produced typical gastric mucosal lesions and haemorrhagical content (massive bleeding) was found in stomach lumens of all rats (6/6) in the control groups (Figure 3). The average ulcer index (UI) of controls was very high (3.33 ± 1.03 in sunflower and palm oil control group, and 3.5 ± 0.5 in olive oil control group). On the other hand, pretreatment with E1, E2 and E3 at a dose of 1.25 mL/kg significantly prevented the gastric mucosal lesions induced by CRS, as compared to the control group (p < 0.05). Percentages of inhibition in UI were 35%, 62% and 40%, respectively. In addition, pretreatment with E2 showed a statistically significant difference in relation to E1 and E3 (p < 0.05). Only gastric mucosal edema and petechiae were seen in almost all (5/6) animals in this experimental group (Figure 4). Haemorrhagical content was noticed in one stomach and it was slightly petechial bleeding.

It is well known that in rats CRS causes gastric lesions by reduction of gastric blood flow, disturbances of mucus and bicarbonate secretion, prostaglandins and nitric oxide. These lesions are also accompanied by increase of histamine synthesis [15] by the significant increase of proinflammatory cytokines, and reactive oxygen species generation. Free radicals formation plays an important role in CRS pathogenesis, due to generation of lipid peroxides, accompanied by impairment of antioxidative enzyme activity of cells [16,17].

The results of the present study demonstrated that all of three St. John’s Wort oil extracts (1.25 mL/kg) offered significant protection against gastric damaging action of CRS in rats, and that this effect was close to that achieved by the one of the most studied anti-ulcer flavonoid, quercetin [18,19]. Doses used in this study were chosen according to the result already published [13]. Namely, quercetin was effective at an oral dose of 8 mg/kg applied as an oil solution supposing that solubilization of quercetin by lipids and emulsifiers, ethanol or their combination allows quercetin to easily solubilize in intestinal epithelial cells enabling its absorption. Besides, it was found that the effects of the different vegetable oils improved the levels of antioxidant defense systems in rat stomach tissues against oxidative damage [20]. Further, it has been reported that quercetin prevents gastric lesions induced by CRS and pylorus ligation, by significant reduction of intragastric concentration of histamine [21]. Also, it is known that quercetin prevents gastric mucosal lesions induced by ethanol [22].

Surprisingly, pretreatment with St. John’s Wort olive oil extract (E2) resulted in significantly higher gastroprotection (Figure 4) in relation to E1 and E3, despite the lowest quercetin content. The results of our experiments provide evidence that the other constituents of E2 as well, including extragens (olive oil) phytochemicals play an additional role in complex gastroprotective activity. It was demonstrated that olive oil phenolics are powerful antioxidants, both *in vitro* and *in vivo* [23,24]. Besides, it was recently reported that olive oil, thanks to its *minor* components, improved antioxidant defense systems in rat stomach [20]. Furthermore, it was shown that phenolic-reach olive leaf extract significantly attenuates CRS induced gastric lesions by possible synergistic effects of its constituents [25]. In our study, one dose of olive oil we used as negative control didn’t provide gastroprotective activity, and we can only presume that synergism between olive oil minor phenolics and St. John’s Wort constituents exists.

## 3. Experimental

### 3.1. Materials

Samples of *Hypericum perforatum* L. were collected during the flowering period on the Rtanj Mountain, Serbia, at the end of June 2009. A voucher specimen (No. 214/09) has been deposited at the Institiute for Medicinal Plant Research "Dr Josif Pančić".

Palm oil was purchased from Olitalia (Forli, Italy), olive oil from Jan Dekker (Wormerveer, Netherlands) and sunflower oil from Sunce (Sombor, Serbia). Quercetin for gastroprotective activity investigations was purchased from Sigma-Aldrich (St. Louis, MO, USA). Reference quercetin HPLC standard was purchased from Carl Roth (Karlsruhe, Germany). Methanol, H_3_PO_4_, water (HPLC grade) and acetonitrile (HPLC grade) were purchased from Merck (Darmstadt, Germany).

### 3.2. Preparation of the Oil Extracts

Three oil extracts (E1, E2, E3) were prepared by maceration of 100 g fresh flowering tops in three different oils (E1-sunflower oil, E2-olive oil, E3-palm oil) exposed to sunlight for 40 days. Five hundred g of each oil extract was obtained [drug:extract ratio (DER) 1:5].

### 3.3. Quantification of Total Hypericins in Plant Material and the Oil Extracts

The content of total hypericins was determined by spectrophotometric method, from three different probe and expressed in hypericin (% g/g) [26]. The extinction of the solution is read at a 587 nm wavelength, in a 1 cm tub, compared to appropriate solvent as a blank. The dosage of hypericin was established with the aid of absorbance measured at 587 nm, the quantity being calculated based on the formula for specific absorbance:

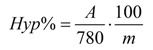

where A = the measured absorbance, m = grams of vegetable product in 100 mL of extract. 780= specific absorbance of hypericine at 587 nm.

For the plant material sample preparation, powdered St. John’s Wort (0.100 g) was extracted for 30 minutes in boiling water to reflux with methanol (100 mL) in a flask with reflux coolers. After cooling, it is filtered and brought to 100 mL with methanol in a volumetric flask. Samples for determination of total hypericins content in St. John’s Wort oil extracts (100 g of E1, E2 and E3) were prepared using the procedure described above.

### 3.4. HPLC Analysis of Plant Material and the Oil Extracts

HPLC analysis of St. John Wort and prepared St. John’s Wort oil extracts was carried out using the same method with Hewlett Packard HPLC model 1090; DAD detector (HP 1040); column Lichrospher RP-18 (5 μm, 250 × 4 mm i.d.) (Merck). The mobile phase A consisted of 99% H_2_O and 1% H_3_PO_4_, while B was acetonitrile. Flow rate was 1 mL/min, and elution was a combination of gradient and isocratic modes: 25–35% B, 0–2 min; 35–60% B, 2–8 min; 60–100% B, 8–12 min; 100% B, 12–25 min.

In the case of plant material, extractive solutions 1% in methanol were used, obtained through the reflux method in water boiling for 30 minutes, in flasks with reflux coolers. Prior to injection, the samples were filtered through a 0.45 μm PTFE filter. The absorption was measured at 360 nm. The injection volume was 20 μL. Identification was carried out thanks to retention time and spectra matching. Once spectra matching succeeded, results were confirmed by spiking with respective standards to achieve a complete identification by means of the so-called peak purity test. Those peaks not fulfilling these requirements were not quantified. Quantification was performed by external calibration with standards.

Samples for the HPLC analysis of St. John’s Wort oil extracts (E1, E2 and E3) were prepared by further reextraction with methanol: a 50 mL volume of each oil extract was reextracted three times with a portion of 50 mL methanol. The methanol extracts were combined, evaporated under vacuum at a temperature below 50 °C and concentrated to a volume of 10 mL. The samples were filtered through a 0.45 μm PTFE filter prior to injection. The absorption was measured at 370 nm. The injection volume was 20 μL. Quercetin was identified by co-injection method, using a commercial standard and its amount in investigated extracts was calculated using calibration curves. Concentrations used for calibration were 0.03-0.5 mg/mL. The results are presented as μg/mL of the oil extract.

### 3.5. Gastroprotective Activity

#### 3.5.1. Animals

Wistar male rats weighing 180–220 g were obtained from Biomedical Research Center, R&D Institute, Galenika a.d. (Belgrade, Serbia). They were housed three per cage under constant environmental conditions (20–24 °C; 12 h light-dark cycle), and were fed with standard pelleted food and water *ad libitum.* This study was performed after approval from the local Institutional Animal Care and Use Committee, and run in accordance to the statements of European Union regarding handling of experimental animals (86/609/EEC).

#### 3.5.2. Gastric Lesions Induction and Evaluation

The animals were randomly divided into seven groups consisting of six rats in each group. They were placed in individual metabolic cages and were fasted for 24 h but with free access to water. Group 1, 2 and 3 represented controls, which received 1.25 mL/kg of sunflower, olive and palm oil respectively intragastricly (i.g.) using metal tube for gavage. Groups 4, 5 and 6 received i.g. 1.25 mL/kg of E1, E2 and E3, respectively, while group 7 represented positive control and received i.g. 8 mg/kg of quercetin dissolved in sunflower oil. After 30 minutes all the groups were submitted to cold-restraint stress (CRS).

For the induction of CRS, the rats were immobilized in individual restraint boxes without possibility of visual contact [27] and kept at 4 ± 1 °C for 3.5 h. This regimen has been reported to produce gastric ulcers reliably in food-deprived rats [28]. Animals were then sacrificed under light ether anaesthesia, their abdomens were opened by a midline incision, and the stomachs were removed, opened along the greater curvature, rinsed gently with water and pinned open for macroscopic examination and for photo-documentation by a digital camera (SONY DSC-H50, Japan). The number and severity of gastric lesions were evaluated according to the scoring system of Buyukcoskun *et al*.: 0 – no lesion, 1 – mucosal edema and petechiae, 2 – from 1 to 5 small lesions (1–2 mm), 3 – more than 5 small lesions or 1 intermediate lesion (3–4 mm), 4 – 2 or more intermediate lesions or 1 gross lesion (greater than 4 mm) and 5 – perforated ulcers [29]. The sum of the total scores divided by the number of animals in group was expressed as the UI ± SD (standard deviation). Percent of inhibition of UI in relation to the control group were estimated from formula:

% inhibition = [1- (UI pretreatment/UI control)] **×** 100

### 3.6. Statistical Analysis

Results are expressed as mean ± SD. Statistical differences between treatments were determined by *t*-test. The level of significance was set at *p* values < 0.05.

## 4. Conclusions

Significant gastroprotection was obtained in experimental model of cold restraint stress in the case of all three oil extracts. Despite the the lowest quercetin content, E2 possessed the highest activity among the investigated samples. Recently performed experiments assumed that quercetin was responsible for gastroprotective activity of oil St. John’s Wort extracts. However, on the basis of our results we can only suppose that content of this flavonoid is important, but not of crucial significance. Hence, we can conclude that other constituents, including those present in oil extracts, as well as in extragens itself, may contribute in gastroprotective activity. Further phytochemical investigations are necessary to provide more evidence in order to precisely determinate which constituent and in what manner influences this pharmacological effect. Also, analyses of oxidative stress parameters in order to determination of mechanism of action are worthy of our further investigations.
